# Evidence of a transnational arts and health practice methodology? A contextual framing for comparative community-based participatory arts practice in the UK and Mexico

**DOI:** 10.1080/17533015.2013.823555

**Published:** 2013-08-08

**Authors:** Anni Raw, Ana Rosas Mantecón

**Affiliations:** a Department of Medicine, Pharmacy and Health, and Department of Anthropology, Durham University, Durham, UK; b Departamento de Antropología, Universidad Autónoma Metropolitana, Mexico City, Mexico

**Keywords:** community arts and health, international comparative research, practice mechanisms, Mexico, ethnographic methodology

## Abstract

**Background:**

This paper draws on new research exploring community-based, participatory arts practice in Northern England and Mexico City to discuss contextual influences on artists’ practice, and whether a common practice model can be identified. The international comparison is used to interrogate whether such a practice model is transnational, displaying shared characteristics that transcend contextual differences.

**Methods:**

The study used multi-site ethnography to investigate the participatory practice of more than 40 artists. Participant observation and extended individual and group dialogues provided data on practice in a diverse range of art forms and settings, analysed using open coding and grounded theory principles.

**Results:**

Findings locate differences in practitioners’ motivations, and perceptions of the work’s function; however, key similarities emerge across both sites, in practitioners’ workshop methodologies and crucially in their creative strategies for catalysing change. A model is presented distilling the key elements of a common practice methodology, found across the study and across art forms.

**Conclusions:**

The discussion notes where divergences echo nationalities of contributors, drawing inferences about the level of influence of national context in this work, and concludes with the implications of these findings for potential international collaboration, to face challenges within the community arts and health sector globally.

## Introduction

Reporting findings from new ethnographic research studying trends in community-based participatory arts and health practice, in the distinct national settings of the UK and Mexico, this paper takes the opportunity for an international dialogue on the research findings. With the authority of first-hand observation, and co-authors from both countries with experience in this field, we frame study findings against the background of differences in the respective contexts in which the work is taking place. We consider in particular, in the light of cultural and contextual characteristics specific to each setting, the significance of the convergences in artists’ practice norms emerging in the study.

The focus of this paper, and the study it seeks to contextualise, is on characterising and contextualising practice, rather than discussing the impacts and outcomes of arts and health projects. As argued in a previous article in this journal (Raw, Lewis, Russell, & Macnaughton, 2012), scholars’ overriding preoccupation with building an evidence base has resulted in community arts and health practice methodology itself remaining a neglected research area. Leaving methods uncharted – perhaps presupposing that artists’ approaches are too individual, diverse, too bespoke to project needs to resonate as a unified narrative – maintains the relative obscurity, even mystique, of arts and health practice (Raw et al., 2012). Such a perspective leaves practitioners isolated, and without a “community of practice” (Wenger, 1998) capable of reflecting on and refining its methodologies (Raw, in press).

Highlighting commonalities and distilling a practice model with a common character, despite immense diversity across the community and participatory arts/health sector even within a single national setting, is a provocative premise. Different definitions for the work and delineations of the sector abound (Badham, 2010; Broderick, 2011; Clift et al., 2009; Dileo & Bradt, 2009; Putland, 2008; Raw et al., 2012; Sonke, Rollins, Brandman, & Graham-Pole, 2009; White, 2010), and diversity of approach is a natural response to diversity of need. To encompass this diversity, a research design using a comparative study of practice methodologies across a wide range of settings offered a broad perspective and the option to contextualise factors that influence different approaches.

We note here the particular value in the opportunity to introduce a fresh comparison of two national contexts for the practice across a wide cultural gulf: one study site in the economically advantaged Global North (UK) and one in a middle-income country in the Global South (Mexico). This offered a high degree of geographic, socio-economic and political distance within the comparison. The two pools of practitioners are working in very different societies, in countries with no shared language, with no directly shared cultural, ethnic or sociopolitical heritage and with few key cultural reference points in common (Beezley, English Martin, & French, 1994). Some cross-fertilisation of ideas between the UK and Mexico in the adjacent field of community development is documented (Pearce, Howard, & Bronstein, 2010); however, no documented or researched sharing of community-based participatory arts practice, or community arts and health work, is known about between the two countries.

In this paper, we thus foreground a comparative study of practice against the contextual backgrounds in which the practice has developed and is currently executed in order to consider the influence of context on the practice itself. The findings as reported in the study are discussed, ultimately considering their implications for the community-based arts/health sectors in both countries.

## Comparative UK and Mexican Definitions for the Work

In terms of arts and health discourses in the contemporary UK context, community-based participatory arts practice is understood as concerning itself with the “social determinants of health” (Marmot, 2005; Marmot, Wilkinson, & Brunner, 2006). Using this socially grounded definition of the causes of ill-health, initiatives that seek, through participatory creative activity, to challenge societal diseases such as social injustice or inequalities, marginalisation, stigmatisation or deprivation easily fall within the “health” frame. Efforts continue at the strategic level to assert and clarify a link between community participatory arts practice and health benefits, with some success at securing resources from the health sector, amidst an increasingly fragmented and piecemeal UK funding picture for the work. As a result of such extensive advocacy by key voices within the sector itself in the UK, the definition “arts and health” is certainly familiar to arts practitioners, and is accepted as one descriptor of this practice.

In the contemporary Mexican cultural context, “health” as a term has strong associations with clinical procedures and medical institutions, with less focus on a social health paradigm. In the past, early post-revolutionary fervour under education minister José Vasconcelos in 1920 saw “Misiones Culturales” using popular theatre to carry health messages to rural populations (Frischmann, 1994); then in the 1960s, the Mexican Social Security Institute (IMSS) promoted cultural activities for its dependents to support citizens’ well-being (the IMSS still maintains the largest theatre network in Latin America (Berman & Jimenez, 2006, p. 89–90)). However, today the links between arts and health are rarely more than implicit. For example, all Mexican correspondents contributing to the study we cite describe clear health benefits from the work: one project leader articulated the purpose of his project using the metaphor of a hospital accident and emergency room (Sebastian, Mexico, 15 November 2011), and participant-observation recorded numerous instances of Mexican participants describing transformative health outcomes from engaging in projects. But since the practice is today rarely framed as specifically health-related, the specific term “arts and health” currently has little resonance in Mexico. Participatory arts activity is more commonly described as “arts education,” or generally linked with wider developmental and social inclusion aims and outcomes than with health (Jimenez, Aguirre, & Pimentel, 2009). This narrowing of “health” concept means that institutional support for community-based participatory arts practice in today’s Mexico resides with cultural and social institutions, and health institutions currently have no connection to this work.

These differences in definitions suggest nuances in understanding of the place of the work within society. However, such nuances may, we argue, be related more to differences in cultural histories and to funding sources and institutional-level policy remits than to more deeply embedded differences in understanding of the work itself. To explore this, we need to reflect briefly on the social contexts for the work in each country.

## Social and Practice Context

### The British Legacy of Radical Movements and Social Concern

Most scholars agree that the historical context for community participatory arts practice in the UK can be traced through successive arts and social movements nationally and internationally throughout the past century. These were groups who believed passionately in using their art form to foster dialogue and create work directly with people, to engage with disenfranchised groups and to give people a voice (Crehan, 2011). Commentators name radical influences across all art forms, especially evident in the international countercultural eruptions of the late 1960s and early 1970s. According to Kelly (1984), artists in the UK were engaged at that time in an “outpouring of apparently radical cultural activity” (p. 9). Hamilton, Hinks, and Petticrew (2003) point to the broad focus of the early community arts movement in the UK as “arts plus social concern” (p. 401) highlighting artists’ preoccupation from the beginning with a wider social change agenda. Further cross-fertilisation of ideas may be traced to the influence of the radical protest movements of this period in the UK, including – of note for this discussion – the feminist tenet “the personal is political,” which became widely absorbed by discourses amongst the alternative left (Hanisch, 1970). The indications from these accounts are that a kind of collective consciousness has developed, amongst artists in the UK, of alternative and participative ways of engaging with communities through their work, and that today’s community participatory arts and health practitioners are clear inheritors of this legacy.

### The Mexican Legacy of Politicised Artists, and State Intervention

Unlike the history in the UK, the Mexican participatory and community arts practice narrative is much less clearly traced. It is important to emphasise that the cultural history of Mexico City itself is specific within Mexico. From the revolutionary period of 1910–1917 onwards, the capital Mexico City weathered a half-century of “cosmopolitan” (as discussed by Suski (2010)) curiosity from the world’s intellectual elite affinity with Mexican post-revolutionary idealism. Mexican muralist Diego Rivera was exported as a powerful Mexican cultural icon, establishing an international identity as a radical political and cultural activist through his mural art, closely aligned with political messaging for the Mexican regime (Marnham, 2000). The tendency for the arts and artists to be seen by governments or communist ideologues in Mexico as their instruments of propaganda was actively resisted by one group of key individuals: muralists O’Gorman, O’Higgins and Morado claimed the necessity of political independence for artists. In 1938 they founded the “Taller de la Gráfica Popular” (people’s graphic arts workshop), which provided top-quality art instruction and production, outside mainstream institutional arts education. Constituting a different form of democratic activism (Azuela, 1993; Azuela, Kattau, & Craven, 1994), this example is perhaps the closest early precursor mentioned in the literature for community-based participatory arts practice in Mexico City. Such activism, a “testimony to (Mexican) artists’ conviction that their work should be at the service of society” (Azuela, 1993, p. 87), is itself a clear theme carrying through to findings from Mexican settings in the central study here. This legacy is seen in the example of the response to the 1985 catastrophic earthquakes in Mexico City, which catalysed a moment of grass-roots activism in response to crisis. From that moment voluntary sector initiatives (some still surviving) sprang up seeking to offer communities – experiencing social exclusion, violence and debilitated social ties – opportunities to engage in cultural activity (Rosas Mantecón, 2011).

The above comparisons of the UK and Mexican community and participatory arts narratives suggest likely differences in the responses of artists to the different pressures and opportunities with which each context confronts them. Using the comparisons to contextualise the research findings in the lead author’s study, we now explore specific findings on convergences and divergences in practitioner perspectives, along lines of national context, and begin here with an outline of the study on which we draw.

## Outline of Central Study

This was a multi-site ethnographic study, the approach combining “anthropology at home” (Rapport & Overing, 2007) at several sites in the north of England, from 2010 to 2012, and an immersive field visit in Mexico City in 2011. Data were generated during extensive participant-observation and semi-structured research dialogues with practitioners. Project participants contributed via a discussion group and other opportunistic dialogues *in situ*. An open coding approach with ongoing thematic analysis supported the inductive interpretation of emerging themes in the data, generated as the research continued (Strauss & Corbin, 1998).

The study focussed on the internal workings of participatory arts practice in community arts and health work – therefore the main research respondents were arts practitioners. Purposively selected for their proven expertise and typicality within the sector, contributors were diverse, including 15 practitioners in Mexico and 26 in the UK, the age range spanning more than 40 years. Twenty-one were male and 20 female; they were from wide-ranging socio-economic backgrounds, with diversity in cultural heritage identities. Highly skilled, trained specialists from more than 20 arts disciplines, what all had in common was their well-regarded current participatory arts practice with groups in non-clinical community settings, in projects seeking change and well-being. Although some have networks in common, there were no pre-existing or current links between the projects or practitioners in the UK and those in Mexico.

Project participants included children; parents with infants; young people, including young offenders in custody; adults facing various health issues; and mixed age groups. Some – families suffering bereavement or experiencing stress in parent–child relationships or experiencing domestic violence – had been referred to a project by a community worker, social worker or doctor. The articulated aims of the projects were as diverse as their settings and formats, and any single project had a range of officially stated aims, attuned to specific, sometimes (especially in the UK) multiple funding sources. The generic objective shared in common was that participants and arts practitioners work together through a creative process in order to address challenges. Challenges such as depression, anxiety, anger management issues or bearing the stigma of a socially marginalised identity or community were common. Often the focus of a project was to confront the range of challenges encountered simply through living or growing up in deprivation: a “social determinants of health” rationale for the work (Broderick, 2011; White, 2009). All projects (in both countries) included in the study were offered to participants free of charge.

## Summary of Study Findings

### Divergences Along Lines of National Context

Certain divergences, across the international comparison in the study’s findings on practitioner discourses, align with themes of difference already highlighted in the comparisons of the UK and Mexican contexts above. The study found some differences between Mexican and British arts practitioners’ personal narratives, tracing the significant experiences that lie behind each individual’s motivation and involvement in this work. These were largely in the accents or intensities of experiences, resulting from the different political and social contexts of the two sites, while many core experiences were similar. For example, though evident in both groups, the number of Mexican respondents recounting previous involvement in political, social and educational activism was higher than that amongst British respondents, and these experiences had carried greater personal risk in the Mexican context. There is a more lifelong direct engagement amongst the Mexican practitioners with the bigger themes of society and state, where they commonly perceived immense and disturbing problems. Mexican practitioners expressed the imperative to challenge systems and norms and to illuminate deep injustices and brutality within Mexican society, here, for example, with reference to the situation of women:

Fighting for these spaces for a multiplicity of voices to be heard I think is crucial. Many women are entirely erased, and have no voice, and are absolutely powerless.

(*Liliana*,^1^*Mexico Dialogue*, *14 November 2011*)

There was a strong sociocultural trend amongst Mexican practitioners’ contributions. They expressed commitment to offering marginalised and disaffected groups within Mexican society access to creative skills, and the chance to learn associated trades. Based on the contributions generated during research dialogues in Mexico, the study characterises these practitioners’ perception of their role as “a socio-cultural and politically engaged proactivity in relation to their society as a whole, working through relationships with individuals and community groups to effect the greater change they perceive to be so urgently needed” (Raw, in press).

The areas in which the British respondents shared more similarities with each other than with the Mexicans were in the personal arena – more British practitioners talked about experiences of marginality and about recognising other individuals’ emotionally challenging situations. Although some expression of political or activist motivation was also evident, the study identifies this as a less bold theme than in Mexico. Trends specific to UK-based practitioner contributions were seen in practitioners feeling driven by a personal desire to collaborate creatively with groups of people, and being motivated by their own curiosity to understand other people and their lives, and the thrill at discovering what people are capable of. British respondents often spoke about the motivating effect of witnessing people change their lives or their outlook:

[Researcher] What keeps you doing it?

I think because I see results of the kind that you never dreamt were possible. You may see somebody come into a room, just in a real state – depressed, and the moment they leave, that mood might have shifted.

(Tony, *UK Group Discussion 5.II*, *1 February 2012*)

Thus, in an echo of the feminist focus on “the personal is political” (Hanisch, 1970) mentioned earlier as influential, the study finds a greater emphasis amongst British practitioners on being motivated by the individual interactions and relationships with project participants and groups (rather than focussing on problems in UK society as a whole). It frames their practice as “seeking through this level of interaction to offer positive inspiration and cultivate (or ‘open up’) possibilities for change, which may take effect at all levels: personal, communal, institutional, cultural, societal, political” (Raw, in press).

Framings of the practice and its agency, both amongst practitioners and within society, seem indeed to be influenced by different national contexts. Hence, we turn to the study’s findings on practice itself, to explore whether these divergences translate into differences in how arts practitioners work with their groups.

### Convergences in Findings – Evidence of a Core Practice

Despite the immense variety of settings, groups with different characteristics and diverse art forms in use, the study traces considerable convergence in the processes arts practitioners engage in their workshops, in both the UK and in Mexico. All engage a set of commonly identifiable elements in their practice, which will be outlined below as a practice “assemblage”^2^; a balanced ecology within the workshop that allows arts practitioners to achieve optimal conditions for an effective project. The assemblage is an ongoing and focussed creative endeavour for them, which can be:

exhausting. It’s peak attention. *Peak* attention.

(*Paula, UK dialogue*, *23 February 2012*)

## An Assemblage of Six Key Elements

Space in this paper constrains the articulation of the practice assemblage to a brief summary; each of its six proposed elements is outlined below indicating its key features only.

### Personal Commitment

Practitioners across the spectrum demonstrated very strong personal motivations for this work, evidencing this as a practice of conviction and often of passion. Some practitioners spoke of feeling driven to be proactive,

giving something back.

(*Ricci*, (*RicciUK Group Discussion 4.II*, (*Ricci11 October 2011*)

I think a lot of people don’t really care, d’you know, sometimes. […] I teach as many kids as I can, and at least they have something, you know because a lot of kids, especially in [city name], don’t have anything, and I can understand that.

(*Lance*, *dialogue*, *UK*, *10 January 2012*)

Many contributors claimed to be “passionate” about this work, and practitioners’ strong commitment was seen manifesting as infectious enthusiasm in their activities, creating an atmosphere of excitement picked up by participants. These are practitioners for whom participatory arts work is a way to express their world view, their convictions and passion for humanity. In the words of Valentina, a puppeteer:

I do this work because it gives me immense pleasure, because I believe that it’s a beautiful way of bringing us closer to each other […]. Because I believe in, and want to pass on this life force, and a passion for life.

(Cecilia, *Email research dialogue from Mexico*, *15 February 2012*)

### Intuition

This universal element is arts practitioners’ self-declared reliance on their ability to function intuitively in the project setting. The study illuminates intuition as a capacity which draws on their imaginative facility (Sennett, 2008); it encompasses the ability of arts practitioners to work responsively:

To intuitively think on your feet …

You go into the room, and you assess it, and then you just pick the thing that’s going to fit, to get you going, and then it builds from there, doesn’t it.

To improvise … so it can come out your finger tips.

(*Ricci*, Dan, Lou *UK Group Discussion 4*, *11 October 2011*)

The study shows reflective imagination feeding intuition in this practice by enabling empathetic sensitivity to others’ vulnerabilities, creating

a super-capacity for empathy

(Mary, *UK dialogue*, *20 January 2010*)

### The Relational Framework

Along with the findings of many other scholars of this practice (Argyle & Bolton, 2005; Davidson & Faulkner, 2010; Everitt & Hamilton, 2003; Kilroy, Garner, Parkinson, Kagan, & Senior, 2007; Macnaughton, White, & Stacy, 2005; Sixsmith & Kagan, 2005; White, 2004, 2009, 2010), this study highlights the central role of relationships in the work. Arts practitioners are shown to co-construct with project participants a framework of highly positive relationships, within which – and only within which – other aspects of the practice can operate effectively. This framework provides a proactive connection through which other developments can take place: first, learning and exploration, with project participants and arts practitioners

learning together […] learning on both sides

(Cecilia, *Email from Mexico*)

Second, framing new experiences (for participants and practitioners alike) such that they can accept the new and the strange, accompanied in these encounters by someone they consider a “friend” (Project participants, UK discussion group, 16 February 2011). Third, relationships built on “unconditional positive regard” (Rogers, 1957) offer project participants’ consistent affirmation, both for who they are and for their efforts. Practitioners’ affirmative gaze invests in participants a confidence to take on personal challenges, and to stretch themselves beyond their own expectations, producing

remarkably brave, challenging work that was a gift to the viewer

(*Paula*, *dialogue*, *UK 23 January 2012*)

The relational framework needs continual effort and attention to the detail of interactions with and between people, and is conceived in the practice assemblage as fluid and dynamic.

### The Spatial Framework

The study shows arts practitioners’ complex spatial practices in this work (cf. Atkinson & Robson, 2012), accessing and shaping “spaces” on three different levels. First, on the literal level, they are adapting physical spaces – the environments in which people meet to participate in creative activities – often establishing a place apart, separate from everyday life. Second, they are working with a collective, affective quality of the group dynamic, often referred to by research contributors as “the space”: the metaphorical space between people and affected by them. Practitioners describe their responsibility for seeking to manage atmospheres as a key aspect of their practice, creating an

environment in which people can flourish

(Ali, *UK, Group Discussion 5*, *1 February 2012*)

The study terms this temporal–spatial dimension the environment of the *dynamic affective atmosphere*, after Anderson (2009). The third spatial dimension, the most closely aligned to artists’ practices (Hyde, 1979), is the “playground” of the practice (Carl, UK Group Discussion 3, 19 August 2011), the world of the creative imagination. This is evident particularly in relation to imagining future realities (Greene, 1995) and to Winnicott’s (1971) “potential space” where play creates a place of collaborative imagination and interaction, neither entirely internal nor of the external world. This multidimensional spatial framework, like the relational framework explained above, requires constant attention, and it too should be understood to be fluid and dynamic, holding and containing the workshop activity.

### The Ethical Framework

This links to the *personal commitment* element outlined above: practitioners in the study explicitly bring their own values, convictions and world views into the environments and processes of their practice by co-constructing with participants a framework of principles and values in which to work together. The common framework is characterised by justice, respect, inclusivity, equality of status, Rogers’ (1957) “unconditional positive regard”, and what one group sums up as simply:

humanity. […]

Well … it’s love really isn’t it!

(Tony *and* Lou A, *UK*, *Group Discussion 5*, *1 February 2012*)

and another:

being decent […] being a decent human being

(Peter, *UK*, *Group Discussion 2*, *20 July 2010*)

Practitioners in both national contexts are proactively fostering these same values and principles as an underpinning foundation of their practice, and working at modelling them in the workshop setting:

It’s where you’re aspiring to be it, as opposed to telling people how to do it.

(Mary, *UK*, *Group Discussion 2*, *20 July 2010*)

As one practitioner described it:

So I guess quite a lot of the work I’m doing … I’ve been thinking in terms of micro-utopias, where it’s possible to really be how we might want to be in the world for about 3 h every week, or 10 min on the street, we can model that and experience it, and in some ways that shifts who and how we see ourselves in the world.

(Ruth, *Skype dialogue*, *3 April 2012*)

### The Creative Key

The sixth, and central, element of the practice assemblage is the element most clearly marking this out as a practice specific to artists, and which draws specifically on their creative expertise. Practitioners are using their creative competencies as stimuli, introducing creative mechanisms and devices within activities, accessing territories common to creative processes and through creative experiences triggering reflexivity for participants to discover new or transformed perspectives on their situation. Arts practitioners’ creative competencies commonly observed included: using imagination; a commitment to artistic quality in the process and its products – essential if the process is to have transformative capacity; and accessing via the artist themselves a quality of difference, or “otherness,” so that perspectives can alter. The creative devices and mechanisms arts practitioners were commonly introducing included:

- metaphor – playing with, juxtaposing and making new meanings in the language of words, dance, music, visual art or any other language;
- absorption, and what Csikszentmihalyi (1991) has conceptualised as “the Flow experience” (p. 40), through which people find a state of “musing,” and an empowering satisfaction, during creative engagement;
- creating something new – usually physically making something, often collaboratively;
- “making special” (Dissanayake, 1980), for example, transforming ordinary spaces, things or experiences into special, extraordinary and precious spaces, things and experiences, heightening the significance and enjoyment of shared experience;
- the subversive mode of playfulness, including laughter, joking (Douglas, 1975, pp. 90–114) and nonsense as a mode, and accessing a collaborative world of play and imagination – playing together – mentioned above in the spatial framework.

The creative territories which the study found projects commonly passing through included risk, and the unknown:

There’s a moment (this usually happens in art) in theatre when you don’t know where to go next. It’s part of the process. What’s the way out? What’s next? and now? What do we do?

(Guillermo, *Dialogue*, *Mexico*, *7 November 2011*)

Even the territory of chaos is often expressly welcomed:

We’re in chaos aren’t we, and change comes from chaos and paradigm shifts come from chaos

(*Lou A*, *UK Group Discussion 5.II*, *1 February 2012*)

Chaos is another heightened reality, as indicated here, closely associated with change and with otherwise unattainable possibilities for renewal or reinvention. Finally, it was common to find shared moments of surrender to the group, and spaces for reflection, which creative processes can facilitate and open up. The study frames these aspects with reference to the potency of Turner’s “communitas” and “liminoid” spaces of ritual (Turner, 1969, 1974, 2002).

Observed across the entire study, evident in both national settings, this complex, interdisciplinary practice assemblage is proposed by the study as a model of community participatory arts practice that transcends national cultural context. This finding raises an interesting disconnect in relation to the impact of contextual variables on its practitioners’ positioning and framing of their work, as outlined above. We now move on to explore this relationship.

## Analysis and Discussion

The study suggests that contextual influences do appear to impact specifically on practitioners’ sense of the place of their work, and hence of their own agency or contribution, within their own societal settings. As we mentioned before, the role of the artist as political and social revolutionary, acting in response to a society in crisis, is a clear historical reference point that helps to explain the identification amongst Mexican practitioner respondents with the notion of the artist as activist. The powerful national narrative of revolutionary artist-activists is a phenomenon resonant in the present, and surely impacting on how Mexican arts practitioners perceive the relevance and agency of their work.

The British historical narrative offers no cultural model that situates artists as iconic revolutionary activists, and maps a society peppered with communities in daily struggle – rather than a society wracked by epic crisis. In contrast to its emergent stage, at which point the practice has been convincingly linked to the political movements of the day (Kelly, 1984), at the current point of its development, the arts funding system in the UK has fragmented any focus on “societal change” into many splintered streams of localised activity and has not promoted overtly sociopolitical leadership by artists at a level of cultural or consciousness change, as in Mexico. Where there is instrumentalisation in the UK arts policy narrative this has latterly been in the service of the softer outcomes of neighbourhood regeneration or health promotion; hence, despite engagement in political activism in their own histories, British community participatory arts practitioners themselves frame their contribution here in terms of its person-centred attributes: a discourse of the personal and communal, as opposed to systemic or structural change.

However, the study demonstrates considerable convergences in the practice itself across all sites. The practice assemblage outlined in this paper – constituting a unifying conceptualisation of a very plural practice – is the unexpected outcome of the study we draw upon here, showing that despite being delivered by very diverse practitioners in entirely different and separate settings in different countries, little of the apparent contextual influence on practitioners carries through into the execution of their work. While descriptors differ, the practice is consistent.

One reason for the similarities in practice could relate to a certain international tide of thought, which may have influenced practice development in both countries. This is the pivotal inspiration of Brazilian Augusto Boal’s (1974/1979) experiments with participatory drama forms through the 1960s in Brazil, and continuing in exile in Europe (Kuppers & Robertson, 2007; van Erven, 2001; White, 2009). Boal promoted theatre as an “open-ended process [… that] shows how the real world can be changed as participants test out their ideas for transforming it” (Frischmann, 1994, p. 294) – a model bearing close resemblance to the practice in the study referenced here. Boal’s work was heavily influenced by the democratic empowerment ideas of his Brazilian contemporary Paolo Freire (1970/1996), working on radical popular education models, which were influential throughout Latin America and beyond (Pearce et al., 2010). Boal and Freire, then, provide an indirect bridge between the participatory community arts ideas and practices developing in the UK and Mexico, as indeed practitioner research respondents in both settings commented.

While acknowledging this one notable unifying influence, we suggest (with a nod to Skinner’s [2007] conceptualisation of a “cosmopolitan” community of practitioners) that this paper has shown that a unifying humanity ensures that the context-specific fluxes in strategic-level interest in the work, demonstrated by policy shifts and changes in the funding landscape, do not impact on the fundamental practice responses of socially engaged artists working with groups who face challenges or crisis. Though there may be different attribution of the purpose or value of the work to different agendas of change and community well-being, in fact the needs of those in the workshop remain consistently rooted in their humanity and in their inability to flourish due to the challenges they face. The study here suggests that the artists’ response through their practice derives from their own “cosmopolitan” humanity such that “their humanity (consciousness, creativity, individuality, dignity) transcend[s] cultural peculiarities” (Rapport, 2007, p. 258). Thus, irrespective of descriptors and funding agendas, their practice draws on whatever creative resources they have, responding with their humanity to help counterbalance those challenges people in their workshops face.

## Conclusion

The counter-intuitive suggestion of this paper that the practice assemblage outlined here may provide an international characterisation of community-based participatory arts (and arts and health) practice (a model that transcends national cultural context) also infers the existence of a “cosmopolitan” (Skinner, 2007) participatory arts/health community of practitioners. The significance of such a finding could be great. Postulating a transnational (or potentially supranational) model for this practice, crucially one that specifically repels contextual and definition differences, could provide a cornerstone for international linking between practitioners and advocates of the work. It could provide a platform for an extended community of practice; this would increase the possibility of shared professional development, and the potential to reflect on and refine practice collectively as an international sector. It would furthermore strengthen the potential for an international alliance for advocacy, and for sustainability solutions. Links between practitioners in different contexts might inspire communities of practitioners in one context to reclaim impetus or directions they have lost through oppressive conditions at home: for example, the political clarity and motivations amongst the Mexican practitioners could inspire British practitioners to reclaim the activist purpose in their work.

The very interdisciplinarity of the practice model could be used to gain visibility and recognition for the practice amongst a wide range of practice fields, and the argument could be made for approaching a diverse collection of sectors for support for the work. Citing the locally recognisable – and yet universal – practice assemblage model could open up avenues for extending institutional support. For example, in Mexico the “health” attributes of the practice could be borrowed from the UK definition to argue for a fresh audience with health institutions at home in the quest for resources. Using an articulation of the model as an “arts and health” practice might prompt Mexican health strategists to connect the links between arts participation and health promotion. The way in which the model incorporates elements from multiple disciplines suggests that it could be used to promote understanding, in both countries as well as more widely, of the multifaceted approach this practice takes towards health promotion agendas. These include connecting effectively with both mental and physical health, independence and resilience, and the dissemination of health messages in innovative ways, amongst many other areas.

Furthermore, the findings discussed here could help stimulate further research of this kind in other countries, both to explore the wider validity and usefulness of this model and to highlight the potential for greater international collaboration in the community arts and health field globally.

## Footnotes

1 All names appearing in this paper in italics are pseudonyms, to protect contributors’ anonymity. Where names appear non-italicised the contributors have expressed a preference to be named.

2 This term is used here not in the “unpredictable” Deleuzo-Guattarian understanding, harnessed by Fox (2012, p.4) in his recent anti-humanist conceptualisation of creativity in relation to health. Here the term is used as a way of describing a purposefully (though findings suggest often intuitively) constructed set of conditions and processes, emphasising the creative hand of the arts practitioner in constructing a balanced ecology of organically interacting elements: the practice assemblage.

## References

[R1] Anderson B (2009). Affective atmospheres. Emotion, Space and Society.

[R2] Argyle E, Bolton G (2005). Art in the community for potentially vulnerable mental health groups. Health Education.

[R3] Atkinson S, Robson M (2012). Arts and health as a practice of liminality: Managing the spaces of transformation for social and emotional wellbeing with primary school children. Health & Place.

[R4] Azuela A (1993). El Machete and Frente a Frente: Art committed to social justice in Mexico. Art Journal.

[R5] Azuela A, Kattau C, Craven D (1994). Public art, Meyer Schapiro and Mexican muralism. Oxford Art Journal.

[R6] Badham M (2010). Legitimation: The case for ‘socially engaged arts’: Navigating art history, cultural development and arts funding narratives. Local-Global: Identity, Security, Community.

[R7] Beezley W, English Martin C, French WE (1994). Rituals of rule, rituals of resistance: Public celebrations and popular culture in Mexico.

[R8] Berman S, Jimenez L (2006). Democracia cultural.

[R9] Boal A (1974/1979). Theatre of the oppressed.

[R10] Broderick S (2011). Arts & Health: An International Journal for Research, Policy and Practice.

[R11] Clift S, Camic P, Chapman B, Clayton G, Daykin N, Eades G, White M (2009). The state of arts and health in England. Arts & Health: An International Journal for Research, Policy and Practice.

[R12] Crehan K (2011). Community art: An anthropological perspective.

[R13] Csikszentmihalyi M (1991). Flow: The psychology of optimal experience.

[R14] Davidson J, Faulkner R (2010). Meeting in music: The role of singing to harmonise carer and cared for. Arts & Health: An International Journal for Research, Policy and Practice.

[R15] Dileo C, Bradt J (2009). On creating the discipline, profession, and evidence in the field of arts and healthcare. Arts & Health: An International Journal for Research, Policy and Practice.

[R16] Dissanayake E (1980). Art as a human behavior: Toward an ethological view of art. The Journal of Aesthetics and Art Criticism.

[R17] Douglas M (1975). Implicit meanings: Selected essays in anthropology.

[R18] Everitt A, Hamilton R (2003). Arts, health and community: A study of five arts in community health projects.

[R19] Fox N (2012). Creativity and health: An anti-humanist reflection. Health.

[R20] Freire P (1970/1996). Pedagogy of the oppressed.

[R21] Frischmann D, Beezley WH, English Martin C, French WE (1994). Misiones culturales, teatro conasupo, and teatro comunidad: The evolution of rural theater. Rituals of rule, rituals of resistance: Public celebrations and popular culture in Mexico.

[R22] Greene M (1995). Releasing the imagination: Essays on education, the arts and social change.

[R23] Hamilton C, Hinks S, Petticrew M (2003). Arts for health: Still searching for the holy grail. Journal of Epidemiology and Community Health.

[R24] Hanisch C (1970). The personal is political. Notes from the second year.

[R25] Hyde L (1979). Some food we could not eat: Gift exchange and the imagination. The Kenyon Review, New Series.

[R26] Jimenez L, Aguirre I, Pimentel LE (2009). Educación artística, cultura y ciudadanía.

[R27] Kelly O (1984). Community, art and the state: Storming the citadels.

[R28] Kilroy A, Garner C, Parkinson C, Kagan C, Senior P (2007). Towards transformation: Exploring the impact of culture, creativity and the arts of health and wellbeing.

[R29] Kuppers P, Robertson G (2007). The community performance reader.

[R30] Macnaughton J, White M, Stacy R (2005). Researching the benefits of arts in health. Health Education.

[R31] Marmot M (2005). Social determinants of health inequalities. The Lancet.

[R32] Marmot M, Wilkinson R, Brunner E (2006). Social determinants of health.

[R33] Marnham P (2000). Dreaming with his eyes open: A life of Diego Rivera.

[R34] Pearce J, Howard J, Bronstein A (2010). Learning from Latin America. Community Development Journal.

[R35] Putland C (2008). Lost in translation. Journal of Health Psychology.

[R36] Rapport N (2007). An outline for cosmopolitan study. Current Anthropology.

[R37] Rapport N, Overing J (2007). Social and cutural anthropology: The key concepts.

[R38] Raw A Durham University, Durham A model and theory of community-based arts and health practice (Doctoral thesis).

[R39] Raw A, Lewis S, Russell A, Macnaughton J (2012). A hole in the heart: Confronting the drive for evidence-based impact research in arts and health. Arts & Health: An International Journal for Research, Policy and Practice.

[R40] Rogers C (1957). The necessary and sufficient conditions of therapeutic personality change. Journal of Consulting Psychology.

[R41] Rosas Mantecón A Projects of creativity and inclusion: The challenges of cultural development in Mexico City.

[R42] Sennett R (2008). The craftsman.

[R43] Sixsmith J, Kagan C (2005). Pathways project evaluation: Final report.

[R44] Skinner J (2007). The salsa class: A complexity of globalization, cosmopolitans and emotions. Identities.

[R45] Sonke J, Rollins J, Brandman R, Graham-Pole J (2009). The state of the arts in healthcare in the United States. Arts & Health: An International Journal for Research, Policy and Practice.

[R46] Strauss A, Corbin J (1998). Basics of qualitative research: Techniques and procedures for developing grounded theory.

[R47] Suski L, Rapport N (2010). Making the cosmopolitan plea: Harold Oram’s international fund-raising in the early cold war. Human nature as capacity: Transcending discourse and classification.

[R48] Turner V (1969). The ritual process: Structure and anti-structure.

[R49] Turner V (1974). Dramas, fields and metaphors: Symbolic action in human society.

[R50] Turner V, Lambek M (2002). Liminality and communitas. A reader in the anthropology of religion.

[R51] van Erven E (2001). Community theatre: Global perspectives.

[R52] Wenger E (1998). Communities of practice: Learning, meaning and identity.

[R53] White M (2004). Arts, mental health and social inclusion. A Life in the Day.

[R54] White M (2009). Arts development in community health: A social tonic.

[R55] White M (2010). Developing guidelines for good practice in participatory arts-in-healthcare contexts. Journal of Applied Arts and Health.

[R56] Winnicott D (1971). Playing and reality.

